# Development of a cost effective three-dimensional posture analysis tool: validity and reliability

**DOI:** 10.1186/1471-2474-14-335

**Published:** 2013-12-01

**Authors:** Yolandi Brink, Quinette Louw, Karen Grimmer, Kristiaan Schreve, Gareth van der Westhuizen, Esmè Jordaan

**Affiliations:** 1 Division of Physiotherapy, Department of Interdisciplinary Health Sciences, Faculty of Medicine and Health Sciences, Stellenbosch University, PO Box 19063, Tygerberg 7505, South Africa; 2 International Centre for Allied Health Evidence (iCAHE), University of South Australia, GPO Box 2471, Adelaide, SA 5000, Australia; 3 Department of Mechanical and Mechatronic Engineering, Faculty of Engineering, Stellenbosch University, Stellenbosch, South Africa; 4 Department of Biostatistics, Medical Research Council of South Africa, PO Box 19070, Tygerberg 7505, South Africa; 5 Statistics and Population Studies Department, University of the Western Cape, Private Bag X17, Bellville 7535, South Africa

**Keywords:** Validity, Reliability, Posture, Adolescents, Three-dimensional measurement

## Abstract

**Background:**

The lack of clear understanding of the association between sitting posture and adolescent musculoskeletal pain, might reflect invalid and/or unreliable posture measurement instruments. The psychometric properties of any new measurement instrument should be demonstrated prior to use for research or clinical purposes. This paper describes psychometric testing of a new three-dimensional (3D), portable, non-invasive posture analysis tool (3D-PAT), from sequential studies using a mannequin and high school students.

**Methods:**

The first study compared the 3D-(X-, Y- and Z-) coordinates of reflective markers placed on a mannequin using the 3D-PAT, and the Vicon motion analysis system. This study also tested the reliability of taking repeated measures of the 3D-coordinates of the reflective markers. The second study determined the concurrent validity and test-retest reliability of the 3D-PAT measurements of nine sitting postural angles of high school students undertaking a standard computing task. In both studies, concordance correlation coefficients and Intraclass correlation coefficients described test-retest reliability, whilst Pearson product moment correlation coefficients and Bland-Altman plots demonstrated concurrent validity.

**Results:**

The 3D-PAT provides reliable and valid 3D measurements of five of the nine postural angles i.e. head flexion, neck flexion, cranio-cervical angle, trunk flexion and head lateral bending in adolescents undertaking a standard task.

**Conclusions:**

The 3D-PAT is appropriate for research and clinical settings to measure five upper quadrant postural angles in three dimensions. As a measurement instrument it can provide further understanding of the relationship between sitting posture, changes to sitting posture and adolescent musculoskeletal pain.

## Background

The association between adolescent sitting posture and musculoskeletal pain is poorly understood [1]. Although research has reported significant associations between postural data and upper quadrant musculoskeletal pain in children and adolescents, the effect sizes and odd ratio’s for these associations were small [1]. The lack of reliable and valid posture measurement instruments, which can be applied with confidence in any setting, underpins the poor evidence base for the association between posture and pain [2]. Current literature also provides evidence for the aetiology of adolescent musculoskeletal pain to be multifactorial in nature and could be attributed to psychological, social and environmental factors [3-5], which adds to the complexity of determining the risk factors for adolescent musculoskeletal pain.

Various three-dimensional posture measurement instruments such as the Elite optoelectronic system (Italy); the FASTRAK electromagnetic digitizer (USA); the Metrecom electromechanical 3D digitizer (USA); the Optotrak motion analysis system (Canada); the Peak Motus motion analysis system (USA); positional MRI; the Qualysis Proreflex Motion Capture Unit system (Sweden); the Vicon T-series motion analysis system (UK) and the Zebris CMS20 ultrasound-based device (Germany) have been used in previous research to assess the sitting posture of children, adolescents or adults [6-14] in a laboratory-based set-up. However since no cost effective, portable, reliable and valid 3D posture measurement instrument was identified in a systematic review of the literature, a new 3D Posture Analysis Tool (3D-PAT) was developed to assess sitting posture of adolescents, in their computer classroom settings [2]. The advantages of the new 3D-PAT is 1) that with estimated cost of US $3 000.00, it is 8–66 times less expensive than other 3D posture measurement instruments e.g. the Elite, Vicon, Zebris, FASTRAK and Optotrak systems; 2) it allows the subject to remain fully dressed compared to rasterstereography which describes the back surface of undressed subjects; 3) portable as it had to be transported to schools to assess students’ sitting posture in their own computer classrooms, while they work on desktop computers which is not possible with e.g. positional MRI which is laboratory-based and 4) is readily configurable allowing it to be adapted to varied classroom computer workstation set-ups (i.e. different dimensions and arrangements) compared to most of the optoelectronic camera system which require ample space for set-up due to the size of the cameras.

Static photographic analysis is currently the most cost-effective, practical and least time-consuming method for measuring several postural angles simultaneously [15]. The 3D-PAT is a basic implementation of stereovision, serving as an early-level instrument upon which further improvements would be made once it had been used in the field.

Psychometric testing of any new instrument should be rigorous, involving consecutive experiments to test the performance of the instrument under different conditions [16]. This paper reports the methodology and findings of validity and reliability testing from two sequential studies involving a mannequin and students.

• The mannequin study was undertaken initially, to establish the concurrent validity of the instrument in measuring the 3D-coordinates of reflective markers placed on a mannequin. This study compared data from the 3D-PAT to data obtained using the Vicon motion analysis system (the reference standard) hereafter referred to as ‘the Vicon system’. This study also reported on instrument (3D-PAT) and operator measurement error by testing the reliability of repeated measures of the 3D-coordinates of the reflective markers. The use of a mannequin eliminated individual subject variability and facilitated a clear early understanding of instrument and operator measurement error, free from issues of sitting posture variability. From an engineering perspective, it was essential that the validity of the coordinate data be determined separately from the postural angle calculation process, as the latter process could have introduced further measurement error.

• The second study using high school students was undertaken to determine the concurrent validity and test-retest reliability of the 3D-PAT’s measurements of nine sitting postural angles.

## Methods

### Ethics

Ethical approval was obtained from the Committee for Human Research of Stellenbosch University. The Western Cape Education Department gave permission to contact schools and students. Written informed consent were obtained from the students and parents / guardians prior to data collection of the student study.

### Study designs

Both studies used correlation and repeated measures designs respectively, to test validity and reliability.

### Study setting

Both studies were performed at Stellenbosch University’s Movement Analysis Laboratory, where the Vicon system is hosted.

### Three-Dimensional Posture Analysis Tool (3D-PAT) (new instrument)

The instrument consists of five synchronised 0.3 MP CMOS FireFly MV – 640 × 480 (Point Grey Research) cameras; 6 mm fixed focal-length lenses (Point Grey Research); a computer with the Windows operating system; two steel cross-bars; two steel clamps; two camera tripods; black cloth and a calibration object.

The two cross-bars were each fitted with the cameras and stood facing each other. Black cloth was draped from the cross-bar downwards to create a uniform backdrop for the photographic images. The cameras could be orientated individually. The system captured 100 synchronised frames from each of the five cameras surrounding the object of interest.

The calibration object consisted of 25 wooden dowels of varying lengths, fastened to a wooden board. Reflective spheres were mounted at the head of each dowel. A black sheaf of paper was inserted diagonally across the wooden board, effectively separating the object into two identical halves. The cameras were able to capture sufficient fiducial markers without moving the object and all the cameras shared the same world coordinate system. The positions in the world coordinate system (i.e. the world points) of the fiducial markers on the object were known, as measured by a coordinate measurement machine. The values were used in the calibration algorithm.

Custom software was written. Input data included images, world points and marker definitions, the number of active cameras, the identifying student names and the capture calibration. The data output were presented in comma-separated values (.*csv*) files. The Point Grey FirePro software development kit incorporated the hardware drivers in order to interface the operating system with the cameras and to synchronise all active cameras [17]. Separate marker placement models were written for the reflective markers of the students (n = 9), mannequin (n = 14) and calibration object.

Vicon motion analysis system (reference system):

The output from the Vicon T-series motion analysis system (Vicon Motion Systems (Ltd) (Oxford, UK), with Nexus 1.4 116 software and giganet communication, represented the reference standard for 3D measurement [18]. Five and eight infrared Vicon T-10 cameras were used for the mannequin and student studies respectively. The system captured 200 frames per second.

### Preparation for validity and reliability testing

The camera unit (cameras, cross-bar, clamp, tripod, and black cloth) of the 3D-PAT was connected to the computer via the IEEE hubs, cabling and the IEEE 1394b Firewire bus expansion card. Two tripods were positioned on either side of the mannequin or student, parallel to the X-plane and frontal plane of the mannequin and student respectively. The cameras were focused and synchronised on the system. The calibration object was captured within the capture volume of the 3D-PAT. Each camera had to be able to capture the entire calibration object during one capture trial and produce a per camera matrix. Separate calibration procedures were performed for the mannequin and students studies. The entire set-up of the instrument took approximately 10 minutes to complete and occurred only once prior to each of the two studies.

**Figure 1:**
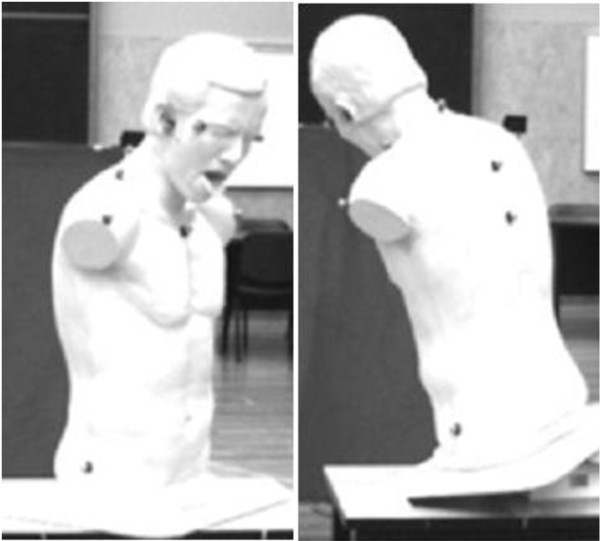
The placement of the reflective markers on the mannequin.

For the mannequin study, the *‘Choking Charlie’ Heimlich Abdominal Thrust Maneuver Training mannequin* was positioned on a wooden table in the centre of both instruments’ capture volume, to ensure that each marker was visible by two or more cameras from each instrument. Reflective markers were placed on both canthi, tragi, acromioclavicular joints, midpoint of shoulders, hips, spinous processes (SPs) of C_7_, T_5_ and T_8_ and the superior border of the sternum, using double-sided tape. Seven different mannequin positions were captured: 1) on a flat surface, facing the Y-plane; 2) on a flat surface, facing the X-plane; 3) on a flat surface, rotated 180° clockwise from the Y-plane; 4) on a flat surface, rotated 135° counter clockwise from the Y-plane; 5) facing the Y-plane, tilted forward; 6) rotated 45° from the Y-plane, tilted forward to the left; 7) facing the Y-plane, tilted forward to the left. Figure 1 illustrates the placement of the reflective markers on the mannequin.

The student study included high school students aged between 15 to 18 years old, from one school. These students were conveniently chosen from a larger student cohort and were already attending the Motion Analysis Clinic to participate in an independent ergonomics study. Sample size calculation was based on a previous study of a similar sample population [19]. An estimated sample size of 31 subjects, with two repeated measurements, level of significance α, and power (1-β) = 95%, was required [20]. Boys and girls studying Computer Application Technology as a school subject were eligible. Students were not excluded if they suffered musculoskeletal pain, however students diagnosed with movement disorders or with severe fixed skeletal abnormalities, were excluded, as this study did not investigate disease or severe postural abnormalities.

**Figure 2:**
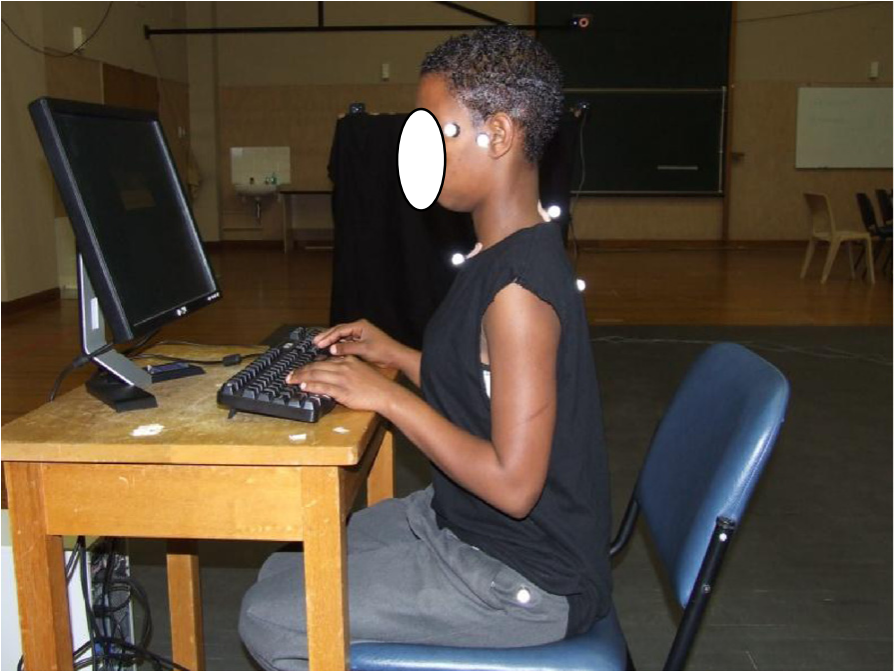
The placement of the reflective markers on a student.

The students wore black t-shirts and grey school pants. Their height was measured with a steel tape measure (Panamedic stature meter) mounted against the wall and their weight with a calibrated digital scale (Terrailon Electronic Scale). Spherical reflective markers, with diameter of 14 mm, were placed on both canthi, both tragi, SPs of C_7_ and T_5_, both greater trochanters and the superior border of the sternum [21]. The markers for the canthus, tragus and greater trochanter were attached to the skin using double-sided tape. Magnets were mounted on the base of the markers for C_7,_ T_5_ and the sternum and were kept in position via a flat magnet plate which was fastened to the skin. This method allowed the students to remain dressed to assure comfort, and support the assumption of habitual sitting posture. The students were given a chair and desk that were similar in height and shape to the furniture used in their school computer laboratory [22]. These were positioned within the capture volume of both instruments. Students were required to sit behind the desk, facing the computer login window (displayed in the centre of the computer monitor) while data were captured. Figure 2 illustrates the placement of the reflective markers on a student.

Nine postural angles were measured [21] and are reported in Table 1 (refer to Additional file 1 for schematic illustrations). The trunk flexion angle describes the thoraco-lumbar spine as one segment and does not differentiate between upper and lower thoracic or lumbar spinal areas. One researcher placed the reflective markers on the mannequin and on all students.

**Table 1:** Definitions of the nine postural angles

**Angle**	**Definition**
**Head flexion (HF)**	The angle between a line drawn from the Cyclops* to the OCI** and the vertical axis.
**Neck flexion (NF)**	The angle between a line drawn from the OCI to the C_7_ SP and the vertical axis.
**Cranio-cervical angle (CC)**	The angle between a line drawn from the Cyclops to the OCI to the C_7_ SP.
**Cervico-thoracic angle (CT)**	The angle between a line drawn from the OCI to the C_7_ SP to the T_5_ SP.
**Trunk flexion (TF)**	The angle between a line drawn from the C_7_ SP to the mid-point of the greater trochanters and the vertical axis.
**Head lateral bending (HLB)**	The lateral angle between a line drawn from the OCI to the tragus, with the vertical line going through the OCI (negative to the left).
**Neck lateral bending (NLB)**	The angle between a line drawn from the OCI to the C_7_ SP, with the vertical axis going through C_7_ in the frontal plane.
**Head rotation (HR)**	The angle between a line drawn from the OCI to the Cyclops, with the anterior axis in the transverse plane (negative to the left).
**Thoracic trunk rotation (TTR)**	The angle between a line drawn from the sternum to the T_5_ SP, with the anterior axis in the transverse plane (negative to the left).

*Midpoint between the left and right canthus.

**Midpoint between the left and right tragus.

**Figure 3:**
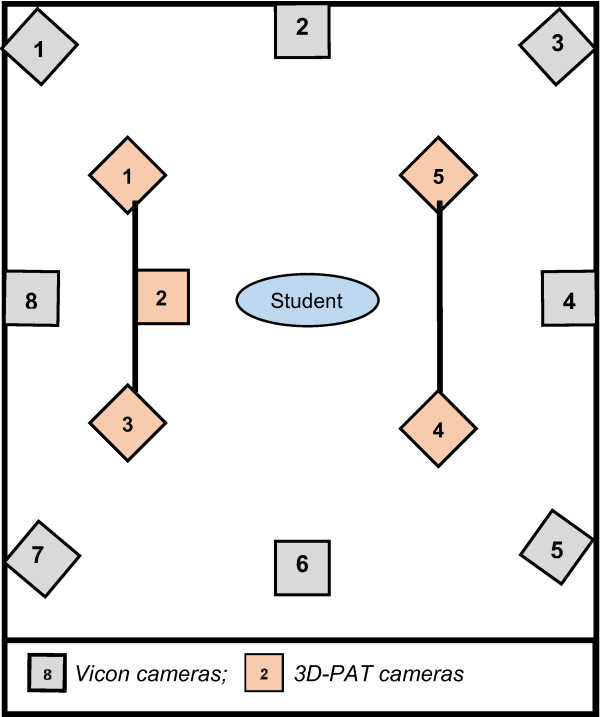
The set-up of the two measurement instruments during the student study.

Figure 3 is a schematic presentation of the set-up of the two measurement instruments during the student study.

### Measurements with the 3D-PAT and Vicon system

The capture trial from each of the measurement instruments commenced simultaneously. Table 2 summarises the measurement procedures for the mannequin and student studies.

**Table 2:** The measurement procedure for both studies

	**Mannequin study**	**Student study**
**Validity testing**	● reflective markers (14) were not removed until the validity testing was complete	● one student measured per trial (27 students)
	● position one was captured three times, and position 2 to 7 was captured once	● data capture commenced once student was settled behind desk
	● capture commenced once the mannequin was in position	● 81 trials in total (27 x 3)
	● nine validity trials in total	
	● 14 X-, Y- and Z coordinate measurements for both instruments	
**Reliability testing**	● two repeated measurements of position one (trials eight and nine)	● for repeated measurements, students were asked to stand after the first trial was captured, and then immediately to sit down, in the same position as previously, before the second trial was captured
	● researcher removed all reflective markers before replacing them, prior to capturing trial eight	● this was repeated for the third measurement
	● same procedure repeated for trial nine, without moving mannequin	
	● no traces of the markers remained before replacing them for the next trial	
	● removal and replacing reflective markers was undertaken to test reliability of operator’s marker placement	

### Data processing of the 3D-PAT

The software program converted all captured images to JPEG format to reduce file size. For each trial, one frame from each camera was selected. The five images were imported into the software program. For validity testing in the mannequin and student studies, and reliability testing for the mannequin, the first frame per camera was selected. For reliability testing in the student study, the first frame per camera per trial was selected, provided that the frames in question resembled a similar posture. If the first frame of a trial did not match the posture from the previous trial, a different frame with closest resemblance to the posture, was selected.

A marker selection procedure was followed according to the marker placement model for the mannequin, student or calibration object to record the image coordinate value for each reflective marker. A square section of the original image containing the desired reflective markers was zoomed and displayed alongside the original image window. In the zoomed window, the centre of each reflective marker was manually selected. The program allowed for a marker not to be selected if it was not clearly visible on the image, and was assigned a ‘none’ value. The image coordinates (image points) and the world point values of the fiducial markers on the calibration object were used together in the calibration algorithm. The coordinate system of the calibration object and the reflective markers on the mannequin or student was the same.

The marker selection files for the mannequin or student trials, along with their respective calibration files, were imported into the reconstruction section of the software program. The image point coordinates and the camera matrix triangulated the 3D-coordinates of each reflective marker. If a reflective marker was not triangulated, the program reported an error message that indicated that the reflective marker was not captured by at least two cameras. The program wrote the coordinates to a text file.

The completed 3D-PAT data set for the mannequin study reported nine sets of data containing the 3D-coordinates of each of the 14 reflective markers. For the student study, the software program automatically calculated the nine postural angles from the 3D-coordinates, using linear algebra and the ‘dot-product-cosine rule’, according to the definition of each angle. A local coordinate system for each subject was defined as follows: from the left to the right greater trochanters defined the *X*-axis, the *Z*-axis was vertically upward and the *y*-axis was perpendicular to the *X-* and *Z*-axes. If all nine postural angles were within acceptable ranges after postural angle calculation, it was regarded a successful trial. The complete 3D-PAT data set for the student study contained 28 and 66 successful validity and reliability trials, respectively. Once the five frames were selected, it took approximately 45 seconds to process the images and produce the values for the nine postural angles per student.

### Data processing of the Vicon system

All the captured frames from each of the mannequin and student study trials were processed to produce 3D-coordinates of the reflective markers. For the student study, the coordinate data were imported into the purpose-built software program of the 3D-PAT, in which the nine postural angles were calculated. The capture frame rate of the 3D-PAT was influenced by the frame rate of the camera sensor and maximum data transfer speed of the connected Firewire bus. Thus the frame of Vicon data, that most closely resembled the postural angles as calculated from the 3D-PAT, was selected for analysis.

### Statistical analysis

**Test–retest reliability** The Concordance correlation coefficient (CCC) with upper and lower 95% confidence intervals (CIs) was calculated for the mannequin study. The index was based on the difference between measurements made by one rater, using one instrument, on two occasions, on the same mannequin position. The Intra-class correlation coefficient (ICC) with upper and lower 95% CI was calculated to measure reproducibility of two or three repeated measurements of the nine postural angles for the student study [23,24]. To judge the strength of agreement, the standards proposed by Landis and Koch [25] were considered.

**Concurrent validity** As a first approach, the measurements from the 3D-PAT and Vicon system were correlated using the Pearson product moment correlation coefficient (г) (PPMCC). The closer the coefficient to -1.00 or +1.00, the stronger the linear relationship and the greater the concurrent evidence of validity of the 3D-PAT [26]. The 3D-coordinates were plotted on scatterplots. The Bland-Altman method allowed calculation of differences between the measurements by the two instruments. This was reported for both studies. The mean difference (***d***) (estimated bias) reflected the systematic difference between the methods; the variation (***s***) about the mean was estimated by the standard deviation (SD) of the differences; and approximately 95% of the differences between the two methods lay between *d* – 1.96 *s* to *d* + 1.96 *s *[27].

## Results

### Mannequin study

The maximum number of coordinates per trial for each set of 3D-coordinates was 14. However, due to the mannequin positioning, not all reflective markers were equally visible in all three planes. Hence, the number of coordinate data sets varied between trials. Four Y-coordinates from trial one were detected as outliers as they were not plotted on, or close to, the 45° line. Trial one was excluded from further analysis.

All other data points were close to, or on, the 45° line, and together with the PPMCC, this indicated that the measures from the 3D-PAT correlated well with the Vicon reference standard (refer to Table 3). Agreement between the measurements from the 3D-PAT and the Vicon system (Bland-Altman findings), is also reported in Table 3. A larger difference was seen for the X-coordinate compared to the Y- and Z-coordinates.

**Table 3:** The validity findings for the X-, Y- and Z-coordinates for trials two to nine (n = 107)

**Scatterplot graphs**	**PPMCC**	**Bland-Altman method**		
	0.95 – 1.00	**Estimated bias**	*Mean difference (d)*	1.98 mm
		**Variation**	*SD of the differences (s)*	10.0 mm
		**CV**	*s/d*	5.07
		**Limits of agreement**	*Upper limit (d + 1.96 s)*	21.64 mm
			*Lower limit (d-1.96 s)*	-17.68 mm
		**Width**	*UL - LL*	39.32 mm
	1.00	**Estimated bias**	*Mean difference (d)*	-0.77 mm
		**Variation**	*SD of the differences (s)*	2.7 mm
		**CV**	*s/d*	3.45
		**Limits of agreement**	*Upper limit (d + 1.96 s)*	4.44 mm
			*Lower limit (d-1.96 s)*	-5.98 mm
		**Width**	*UL - LL*	10.42 mm
	0.98 – 1.00	**Estimated bias**	*Mean difference (d)*	0.71 mm
		**Variation**	*SD of the differences (s)*	1.9 mm
		**CV**	*s/d*	2.73
		**Limits of agreement**	*Upper limit (d + 1.96 s)*	4.51 mm
			*Lower limit (d-1.96 s)*	-3.09 mm
		**Width**	*UL - LL*	7.60 mm

The CCCs for the X-, Y- and Z-coordinates were 0.99, indicating strong reproducibility for all three coordinate systems.

### Student study

Thirty-six students provided signed informed consent for this study. Three boys and six girls were excluded as being too young or old for the required study age range, leaving 27 participants. This provided power of at least 80%. Due to technical problems, the data from 24 students (10 males and 14 females) could be used as only 28 validity trials from ten students and 66 reliability trials from 24 students, were successful. The mean age for the validity study group was 16.2 years (SD 0.8); the mean height 1.63 m (SD 0.1); and the mean weight 54.6 kg (SD 7.7). The mean age for the reliability study group was 16.1 years (SD 0.8); the mean height 1.58 m (SD 0.2); and the mean weight 56.6 kg (SD 11.1).

The mean scores for the first, second and third measurements (means 1–3), are reported in Additional file 2. The PPMCC for head flexion (HF) (0.97; 0.96; 0.99), neck flexion (NF) (0.95; 0.99; 0.98), cranio-cervical angle (CC) (0.90; 0.96; 0.87) and trunk flexion (TF) (0.99; 0.98; 0.98) indicated that the measurements from the two instruments were well correlated for each repeated measurement. For cervico-thoracic angle (CT) (**0.17**; 0.96; 0.78), head lateral bending (HLB) (0.90; 0.97; **0.52**), neck lateral bending (NLB) (0.84; 0.79; **0.57**), head rotation (HR) (0.84; 0.81; **0.63**) and thoracic trunk rotation (TTR) (0.84; **-0.09; 0.34**) at least one of the PPMCC was below 0.7, indicating a poorer correlation. The agreement between the measurements from the two instruments for the nine postural angles is shown in Table 4. The largest positive differences were seen for CT, NLB and CC and the largest negative differences for TTR and HR. The limits of agreement were the widest for CT, NLB and HR. The Bland-Altman plots, demonstrating the agreement between the measurements from the two instruments, are shown in Additional file 3.

**Table 4:** The validity (n = 28) and reliability (n = 24) findings for the nine postural angles

			**HF**	**NF**	**CC**	**CT**	**TF**	**HLB**	**NLB**	**HR**	**TTR**
**Validity**	**Estimated bias**	*Mean difference (d)*	-0.74°	-0.64°	2.65°	3.69°	-0.83°	0.01°	3.57°	-2.70°	-3.93°
	**Variation**	*SD of the differences (s)*	1.96	1.68	3.95	9.24	1.09	1.84	8.78	5.19	3.25
	**Limits of agreement**	*Upper limit (d + 1.96 s)*	3.10	2.65	10.39	21.80	1.31	3.60	20.78	7.48	2.44
		*Lower limit (d-1.96 s)*	-4.58	-3.93	-5.10	-14.42	-2.96	-3.59	-13.64	-12.88	-10.29
	**Width**	*UL – LL*	7.68°	6.58°	15.49°	36.22°	4.27°	7.19°	34.42°	20.36°	12.73°
	**ICC**		0.86	0.69	0.64	0.37	0.78	0.54	0.45	0.29	0.38
	**Student variability**		56.75	26.11	61.81	71.10	26.15	10.69	153.63	12.87	80.12
**Reliability**	**Error variability**		8.86	11.29	32.37	111.79	7.04	8.71	176.26	28.05	120.70
	**Lower 95% CI**		0.76	0.51	0.43	0.08	0.64	0.29	0.18	0.00	0.11
	**Upper 95% CI**		0.96	0.87	0.86	0.66	0.92	0.78	0.72	0.61	0.66

Table 4 also reports the ICC, student and error variability and the 95% CIs for each postural angle. The results indicate almost perfect reproducibility for HF and TF; substantial reproducibility for NF and CC; moderate agreement for HLB and NLB; and poor reproducibility for CT, HR and TTR.

## Discussion

This new tool, the 3D-PAT, offers a novel and persuasive way of measuring 3D adolescent sitting posture in a valid and reliable manner, relevant to a range of research, clinical and workplace settings. The 3D-PAT is portable, inexpensive, and simple to set-up and operate. Potentially it can enhance our understanding of the variability of adolescents’ spinal posture in real-life environments, such as classroom desk activities, or computer screen-based activities at school or home. This paper outlines the processes and results of two consecutive steps taken to test validity and reliability of the 3D-PAT. The mannequin study (which removes the issue of subject variability) demonstrates good to excellent reliability and validity of the new instrument. The second study, which addressed subject variability, demonstrated that the 3D-PAT has a robust capacity to reliably measure four sitting postural angles (HF, NF, CC, and TF) and an acceptable ability to measure HLB of high school students using the same equipment and task in the same environment. Thus differences in repeated measurement of postural angles in adolescents can be attributed to subject variability of static sitting posture, and not errors from the instrument or the operator.

However the angles that reflected movement in the transverse (HR and TTR) and frontal planes (HLB and NLB) were less reproducible than the angles reflecting movement in the sagittal plane (refer to Table 4). Rodacki et al [28] acknowledge that the head has more degrees of freedom than the rest of the spine, which necessarily results in greater variability of movement in the head and neck segment. Thus, the mobility of the head/neck segment could have produced greater variability in the transverse and frontal planes than in the sagittal plane.

Limited literature is available on the reproducibility of any method of measuring adolescent sitting posture, especially where measurement differences pertain to individual variability, and not to marker placement, different raters, testing procedure or instrument. To our knowledge, only Perry et al [15] and Van Niekerk et al [29] describe the reliability of sitting postures in adolescents. Perry et al [15] reported moderate to good reliability for all angles, except HF (ICC = 0.37) and CC (ICC = 0.40), whereas Van Niekerk et al [29] reported very good reliability for head, cervical and thoracic angles (ICC = 0.78-0.97). The only study we found, that measured 3D sitting posture, reported moderate ICCs for seated thoracic curvature (0.69) and lumbar curvature (0.52) of children (adolescents were not tested) [14]. McEvoy and Grimmer [30] suggest that children and adolescents have less ability to resume a required posture, due to anthropometric and motor control immaturity.

The difference in the capture rate of the two instruments also exaggerated sitting posture variability, as there was an increased chance that the instruments failed to capture the exact same sitting posture. However the angular data from the 3D-PAT were matched with a frame from the Vicon system with the best-fitting angular data to compensate for the measurement problem, and therefore we believe that this issue has been addressed.

Poorer validity and reliability scores for the cervico-thoracic angle and thoracic trunk rotation (refer to Table 4) could possibly be due to poor marker reconstruction of the T_5_ SP. A prominent factor that can influence the accuracy of marker coordinate calculation when using cameras, is the angle between the line of sight from two cameras to the marker [31]. The line of sight can vary, depending on the accuracy with which the centre of the spherical reflective marker is located. If the angle between the line of sight from two cameras is small (closer to 0° for calculation of the x-coordinate data, where two cameras are positioned on one cross-bar) or large (closer to 180° for the T_5_ SP digitisation, where two cameras are positioned on either side of the student), a small deviation in the orientation of the line of sight produces a large error in calculating the depth (the distance from the camera) of the coordinates. In our study, often only half the T_5_ marker was visible, thus compromising the accuracy in depicting the centre of the marker, which also influenced the orientation of the line of sight.

**Figure 4:**
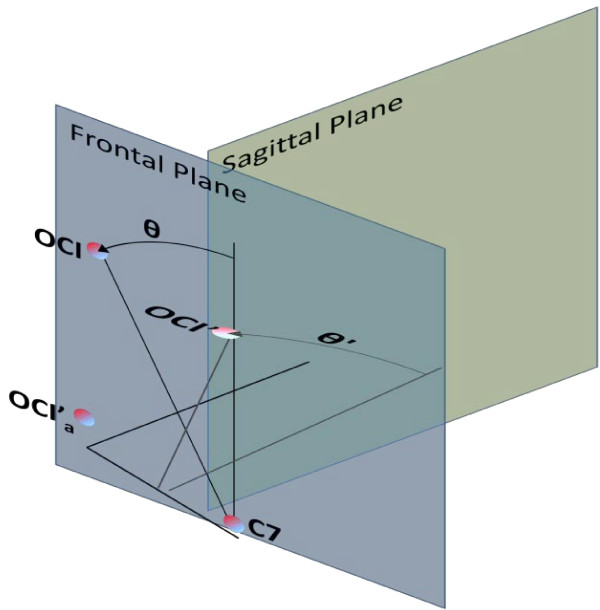
The projection fault occurring when NLB was calculated in the frontal plane.

The student study found mean sitting postural angles for adolescents within the ranges reported in studies that used the same angle definitions [10,32-35], for instance HF (66° to 80°), CC (151° to 160°), CT (149° to 152°), TF (-13° to -22°), NLB (0.4° to 0.9°) and HR (-0.4° to 1.7°). No other study reported HLB or TTR as defined in our study, thus comparison could not be made with the previous research. However, the variation, as demonstrated by the SD of NLB (±12.4°) and HR (±7.6°), was greater in our student study when compared to a study by Straker et al [10], which reported an SD of ±0.9° for both the angles concerned. The greater variability in NLB could be due to a projection fault, which may have occurred when NLB was calculated in the frontal plane. There are two ways in which NLB, represented by the θ angle in Figure 4, could be measured. In the first case, the OCI moved only in the frontal plane and θ was measured in the frontal plane. Thus, the angle was a true reflection of NLB. In the second case, the same magnitude of OCI movement laterally was also accompanied by neck flexion in the sagittal plane, so that the OCI’ was no longer within the frontal plane. However, the θ angle was still measured in the frontal plane once the OCI’ was projected onto the frontal plane, which was represented by OCI’_a_. The θ angle was measured between the vertical line and OCI’_a_ which was not a true reflection of NLB in the frontal plane. We suggest that NLB be defined as the smallest angle between a line from the OCI to the C_7_ SP in the sagittal plane. HR was consequently also influenced by NLB.

## Conclusions

The new posture measurement instrument (3D-PAT) is portable, low cost, user friendly, valid and reliable. Of the nine postural angles tested, we found that it provided robust and acceptable measures of four (HF; NF; CC; and TF) and one (HLB) sitting postural angles in three dimensions respectively. It was tested on students under the same sitting task and environmental conditions. Currently this instrument could be used to measure 3D adolescent posture, in terms of five upper quadrant postural angles, in clinical and real-life settings, and may therefore assist in establishing a better understanding of posture in adolescents. Better real-life posture measurement may improve the understanding of the relationship between posture and musculoskeletal pain.

## Abbreviations

3D: Three-dimensional; 3D-PAT: Three-dimensional posture analysis tool; csv: Comma-seperated values; SPs: Spinous processes; ICC: Intra-class correlation coefficient; CCC: Concordance correlation coefficient; CIs: Confidence intervals; PPMCC: Pearson product moment correlation coefficient; SD: Standard deviation; CV: Coefficient of variation; HF: Head flexion; NF: Neck flexion; CC: Cranio-cervical angle; CT: Cervico-thoracic angle; TF: Trunk flexion; HLB: Head lateral bending; HR: Head rotation; NLB: Neck lateral bending; TTR: Thoracic trunk rotation.

## Competing interests

All authors declare that they have no competing interests.

## Authors’ contributions

All the authors contributed to the conception and design of the study, two authors (YB and GvdW) acquired the data, two authors analyzed the data (EJ and YB), all the authors contributed to the interpretation of the data, three authors (YB, QL and KG) drafted the manuscript and all the authors critically appraised the content of the manuscript. All authors read and approved the final manuscript.

## Pre-publication history

The pre-publication history for this paper can be accessed here:

http://www.biomedcentral.com/1471-2474/14/335/prepub

## Supplementary Material

Additional file 1 Schematic presentation of the nine postural angles. Click here for file

Additional file 2 The mean and SD for the repeated measurements from the 3D-PAT (n = 23/22/21). Click here for file

Additional file 3 Bland-Altman plots for the nine postural angles demonstrating the agreement between the measurements from the two instruments. Click here for file
