# Supplementary figures and images for: Development of a cost effective three-dimensional posture analysis tool: validity and reliability
Source: BMC Musculoskelet Disord. 2013 Dec 1;14:335. doi: 10.1186/1471-2474-14-335 (PMC4219581; doi:10.1186/1471-2474-14-335)

Supplementary file 1: Schematic presentation of the nine postural angles


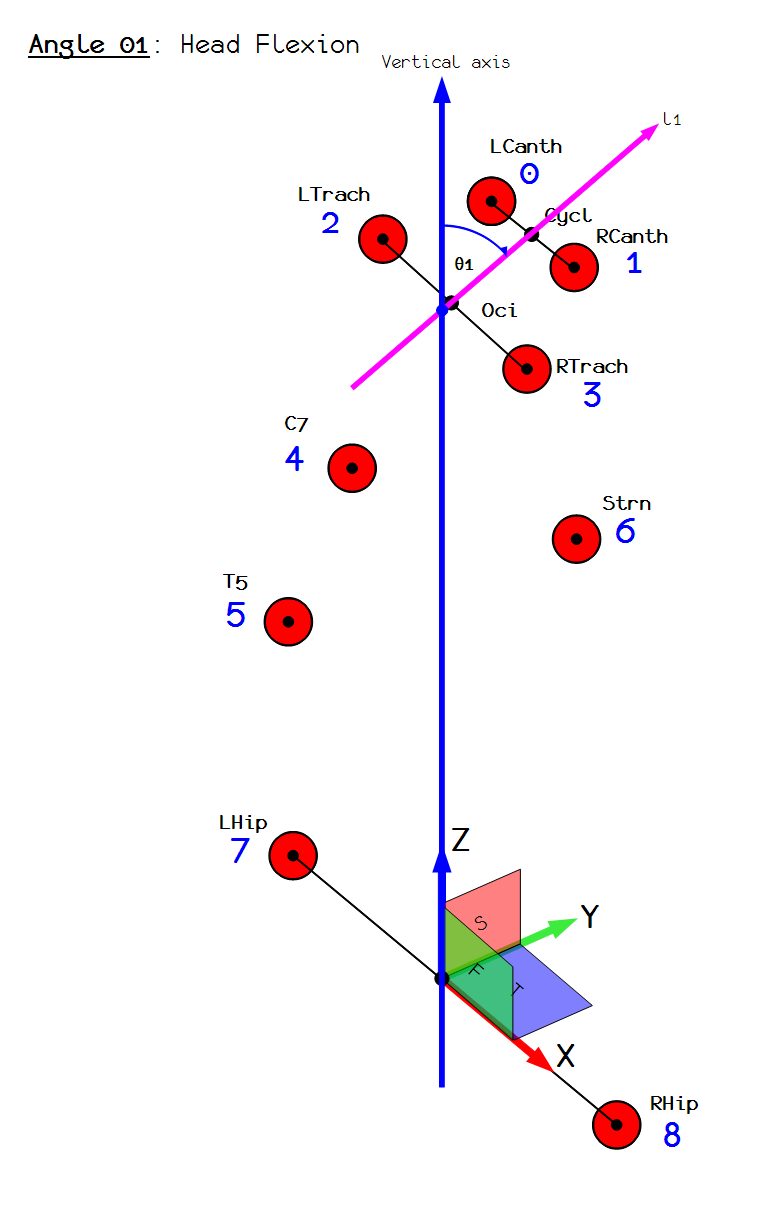

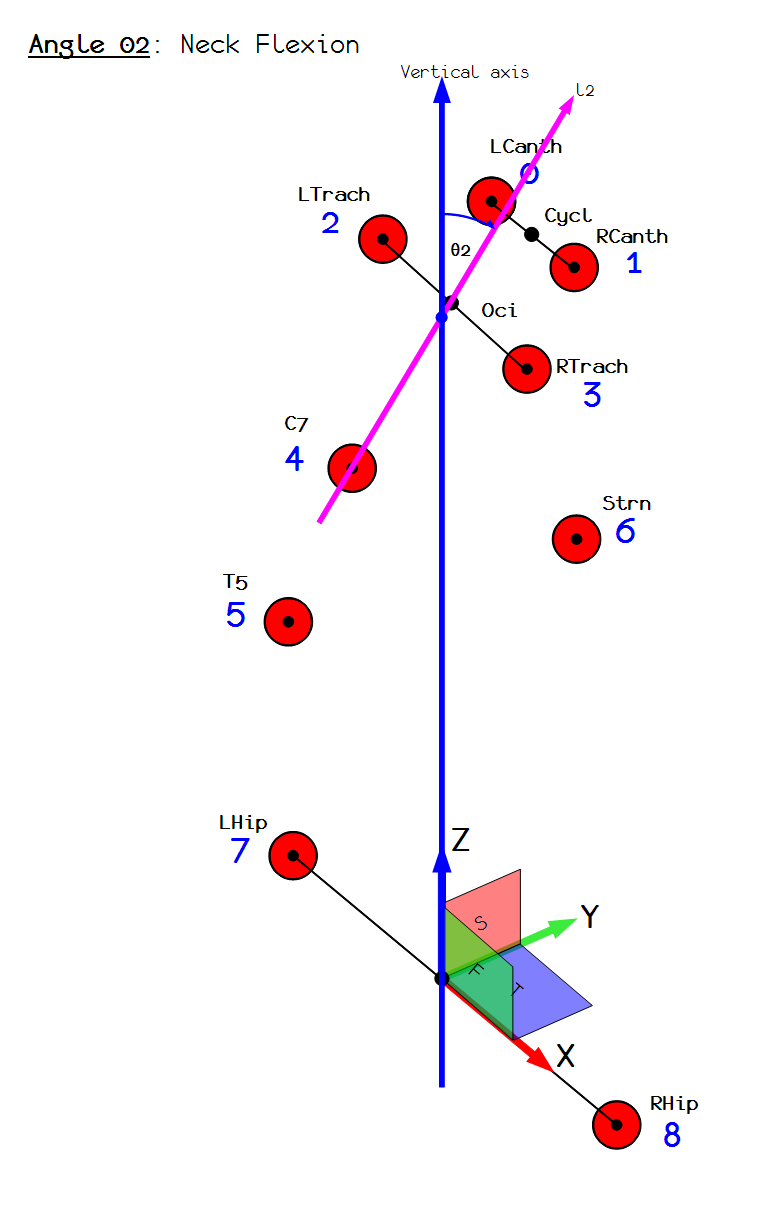

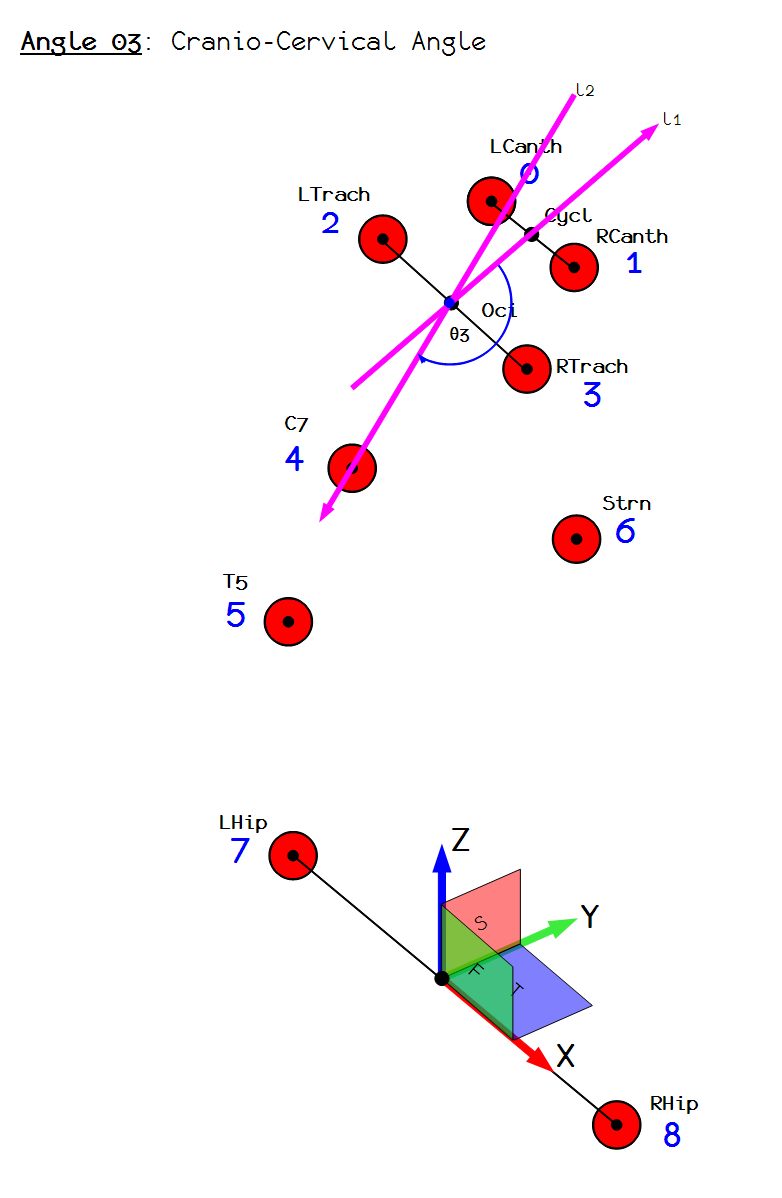


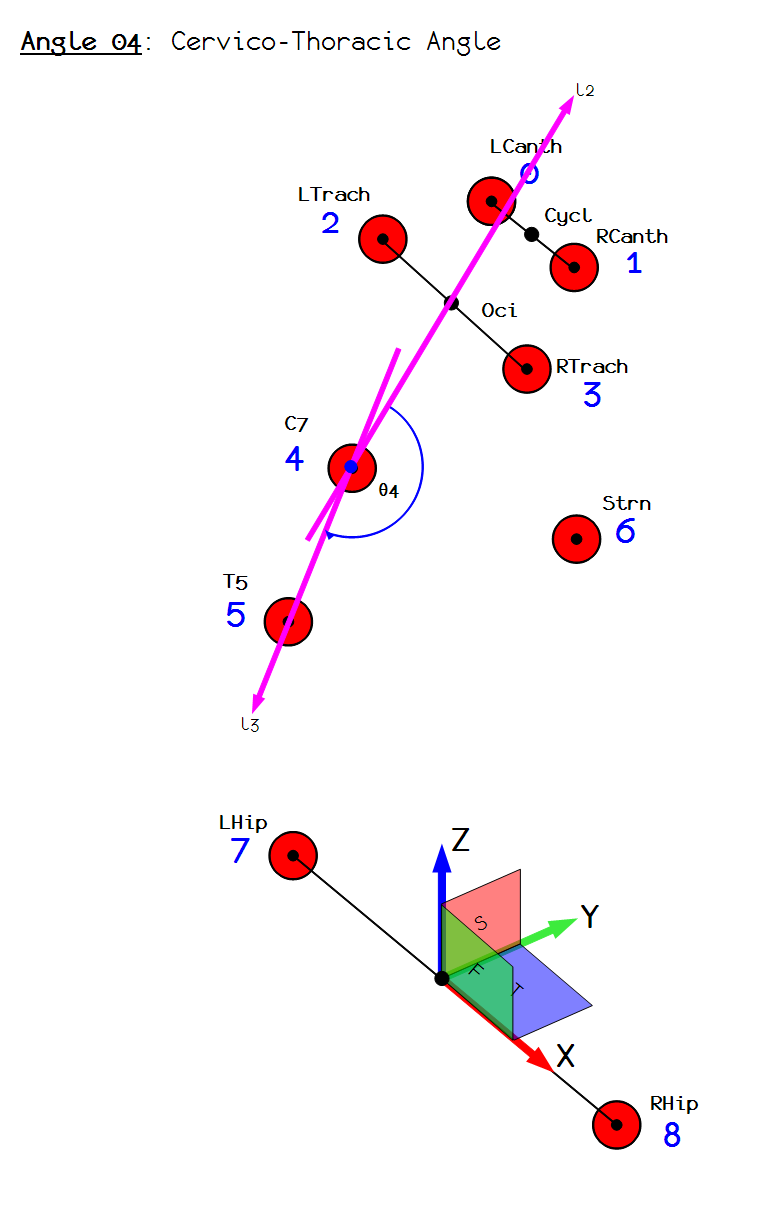

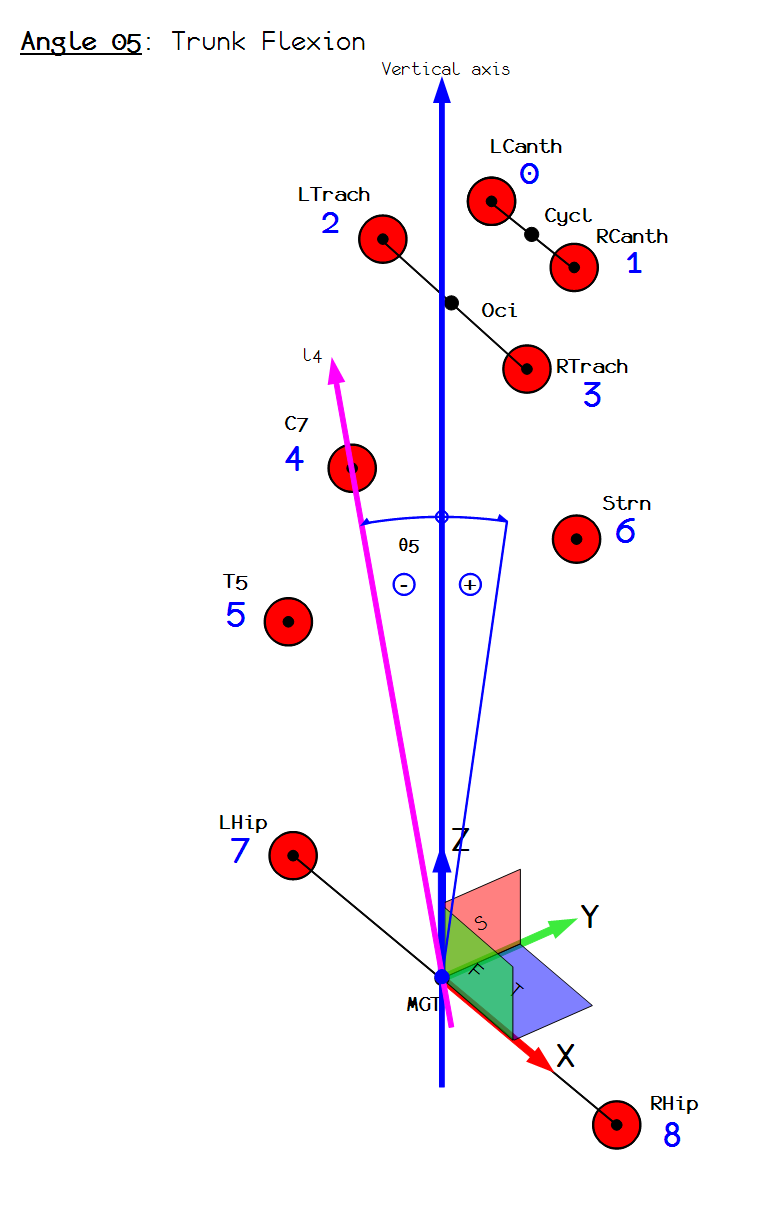

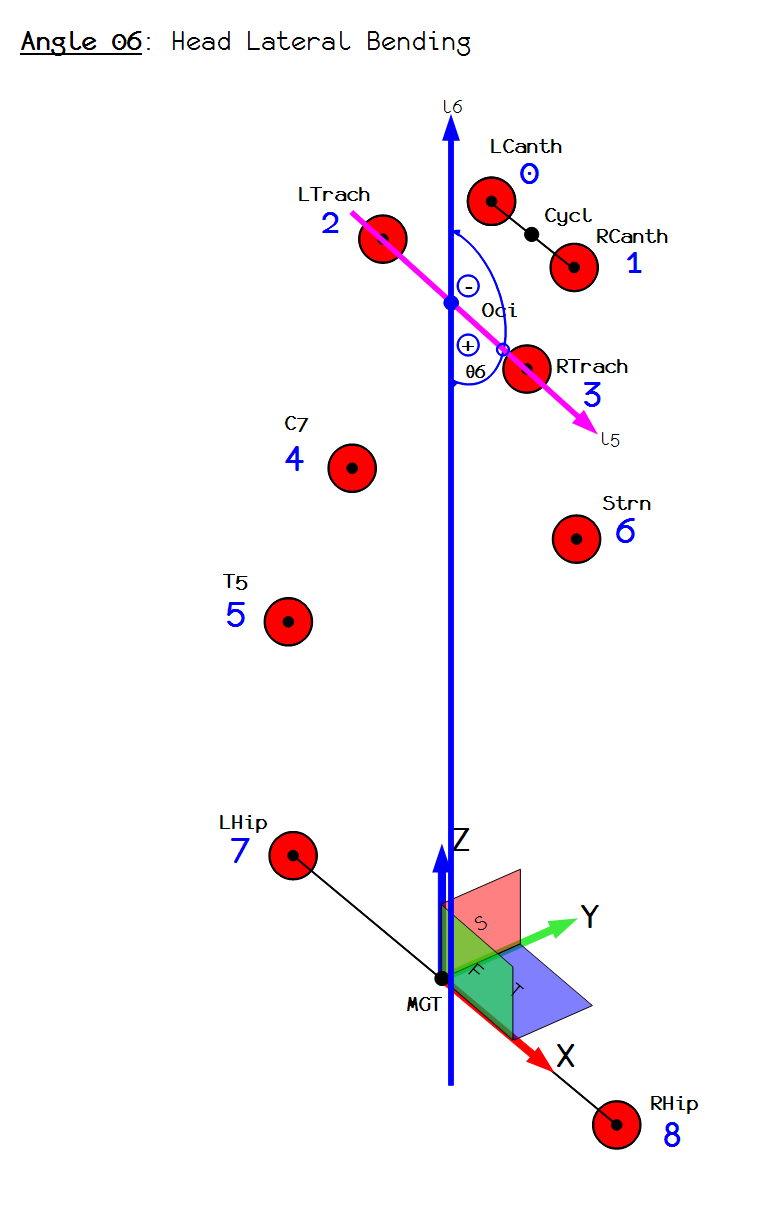


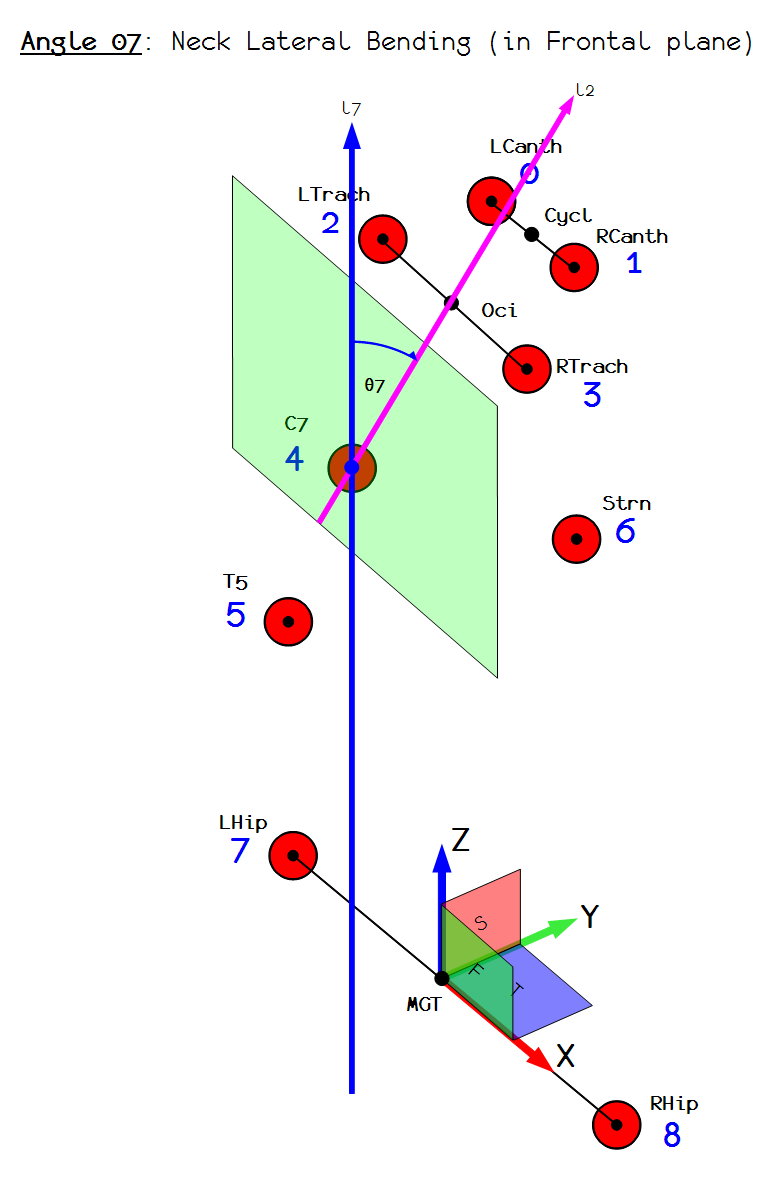

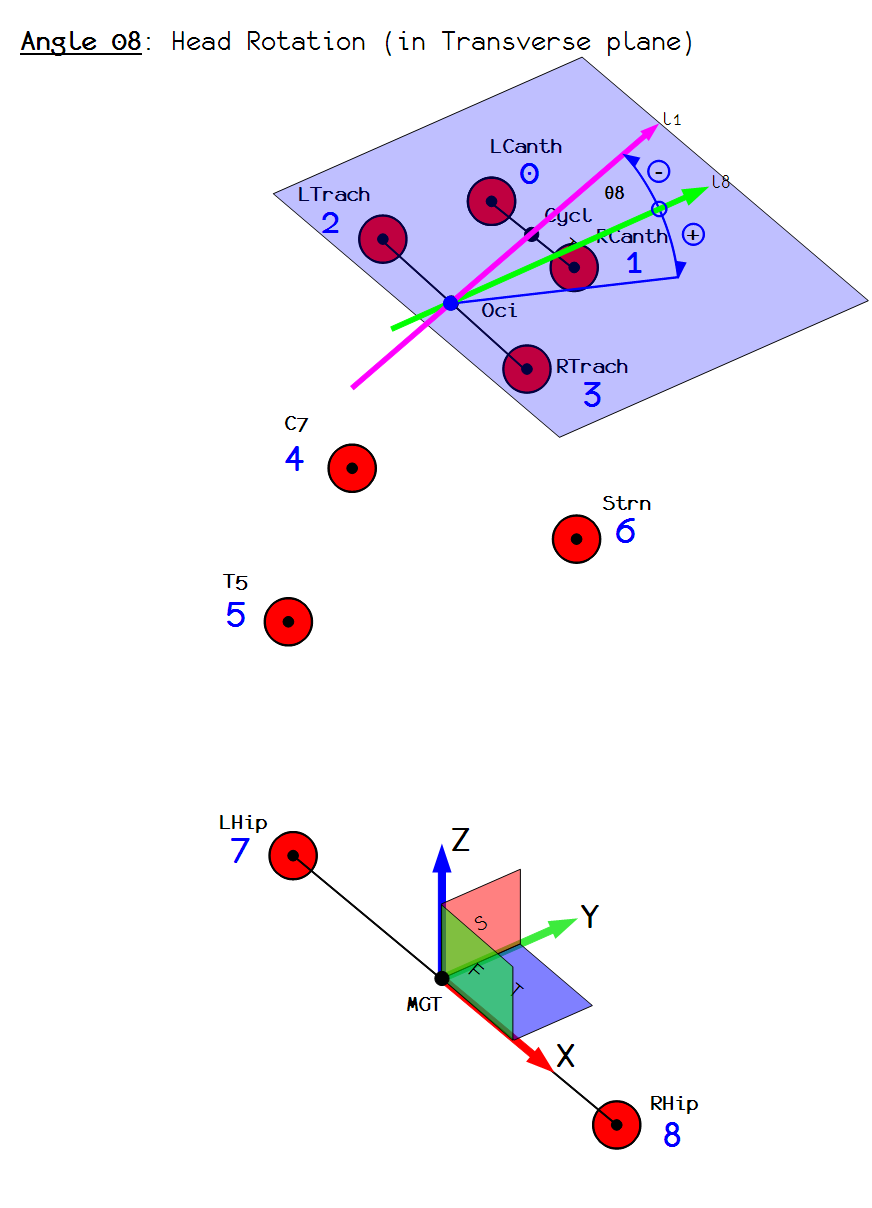

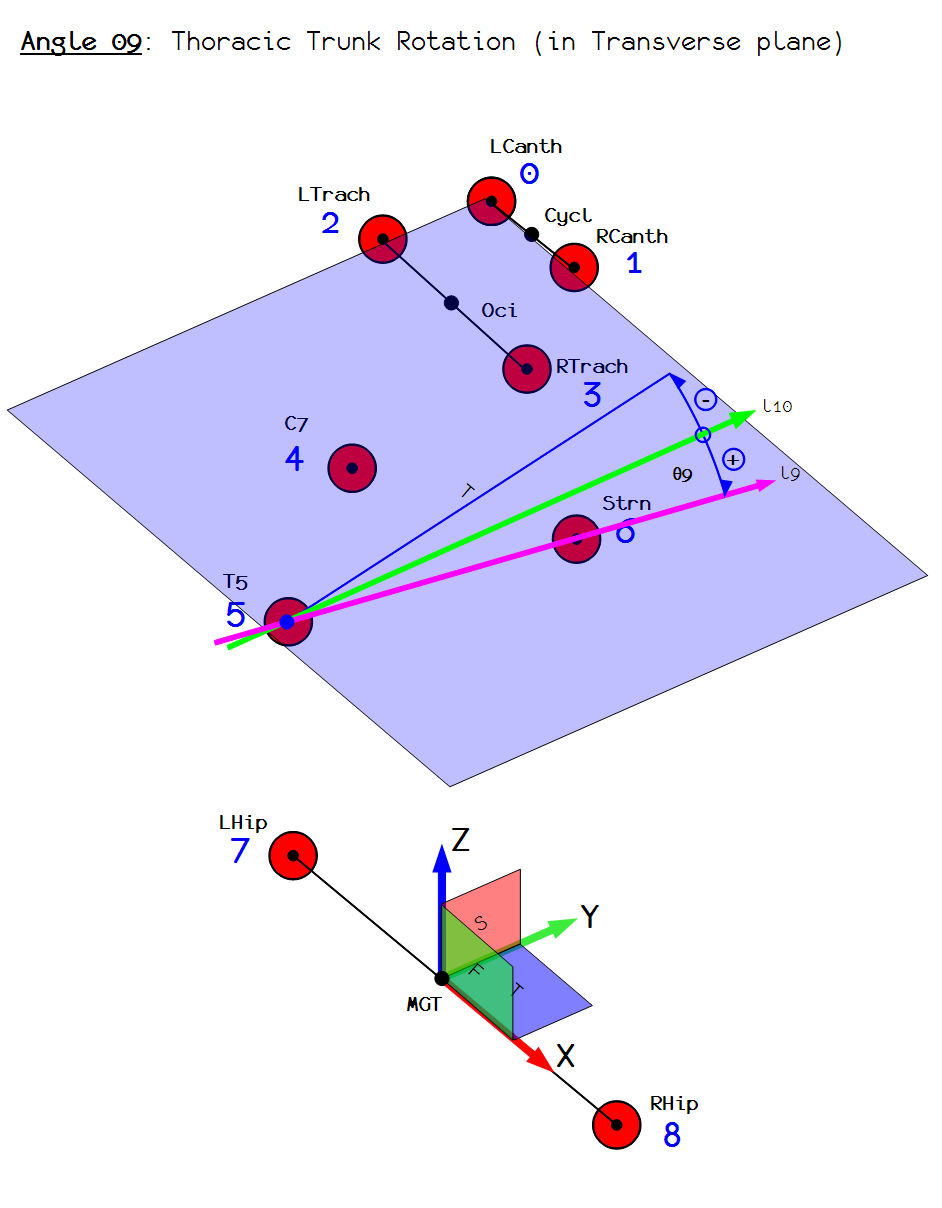

Supplement: Additional file 1 — Schematic presentation of the nine postural angles. [file 1471-2474-14-335-S1.docx]
