# Supplementary material for: Development of a cost effective three-dimensional posture analysis tool: validity and reliability
Source: BMC Musculoskelet Disord. 2013 Dec 1;14:335. doi: 10.1186/1471-2474-14-335 (PMC4219581; doi:10.1186/1471-2474-14-335)
Supplement: Additional file 2 — The mean and SD for the repeated measurements from the 3D-PAT (n = 23/22/21). [file 1471-2474-14-335-S2.docx]

Supplementary file 2: The mean and SD for the repeated measurements from the 3D-PAT (n = 23/22/21)

|  | **Mean 1 (°)**  **n = 23** | **Mean 2 (°)**  **n = 22** | **Mean 3 (°)**  **n = 21** |
| --- | --- | --- | --- |
| **HF** | 68.9 (±7.5) | 69.9 (±7.5) | 66.6 (±8.1) |
| **NF** | 57.0 (±6.2) | 56.4(±6.46) | 55.8 (± 6.3) |
| **CC** | 160.8 (±8.6) | 159.4 (±9.6) | 158.3 (± 10.6) |
| **CT** | 150.9 (±12.8) | 152.9 (±10.5) | 148.8 (± 16.7) |
| **TF** | -8.5 (±4.0) | -9.4 (±7.0) | -8.4 (± 5.3) |
| **HLB** | 4.8 (±4.6) | 4.9 (±5.2) | 3.2 (± 2.9) |
| **NLB** | -12.7 (±18.3) | -13.2 (±17.8) | -17.4 (± 18.6) |
| **HR** | 3.2 (±6.0) | 1.2 (±6.8) | 3.4 (± 6.2) |
| **TTR** | 0.3 (±11.1) | -9.4 (±17.7) | -2.4 (± 11.4) |
