# Supplementary material for: Development of a cost effective three-dimensional posture analysis tool: validity and reliability
Source: BMC Musculoskelet Disord. 2013 Dec 1;14:335. doi: 10.1186/1471-2474-14-335 (PMC4219581; doi:10.1186/1471-2474-14-335)
Supplement: Additional file 3 — Bland-Altman plots for the nine postural angles demonstrating the agreement between the measurements from the two instruments. [file 1471-2474-14-335-S3.docx]

Supplementary file 3:
